# The Composition of Subgingival Microbiome in Hidradenitis Suppurativa and Periodontitis Patients

**DOI:** 10.3390/pathogens12030377

**Published:** 2023-02-25

**Authors:** Beata Jastrząb, Barbara Paśnik-Chwalik, Katarzyna Dębska-Łasut, Tomasz Konopka, Piotr K. Krajewski, Jacek C. Szepietowski, Łukasz Matusiak

**Affiliations:** 1 Department of Dermatology, Venereology and Allergology, Wroclaw Medical University, 50-368 Wroclaw, Poland; 2 Department of Periodontology, Wroclaw Medical University, 50-425 Wroclaw, Poland

**Keywords:** hidradenitis suppurativa, periodontitis, subgingival microbiome, oral microbiota, periodontal health

## Abstract

Hidradenitis suppurativa (HS) is a chronic inflammatory disorder of the pilosebaceous unit of the intertriginous body areas. Recent findings have suggested the association between periodontitis and HS. This investigation aimed to characterize and compare the composition of subgingival microbiome between HS, periodontitis, and control patients. The nine crucial perio-pathogenic species and total bacteria were analyzed using RT-PCR based tests in samples collected from 30 patients with periodontitis, 30 patients with HS and 30 controls. Patients with HS were excluded if they had periodontitis and patients with periodontitis were excluded if they had HS. The mean total bacteria count was significantly higher in HS and periodontitis samples than in control samples (*p* < 0.05). The majority of perio-pathogens tested were more frequently detected in HS and periodontitis groups than among controls. *Treponema denticola* was the most common pathogen in individuals with HS (70%) and periodontitis (86.7%), while among controls *Capnocytophyga gingivalis* was the most frequently detected isolate (33.2%). The results of the present investigation demonstrated that HS and periodontitis patients share some similarities in their subgingival microbiome composition.

## 1. Introduction

Hidradenitis suppurativa (HS) is a chronic inflammatory dermatosis that is characterized by deep-seated nodules and abscesses that rupture and lead to sinus tracts formation and scarring. The estimated prevalence of HS ranges from under 1% to 4%. These numbers may be underestimated as underdiagnosis or inadequate diagnosis are common events. The age of onset of the disease is usually between puberty and the fourth decade of life, most commonly from age 21 to 29 [1]. This condition most commonly involves the intertriginous skin of the axillary, inguinal, inframammary and genital regions of the body [2]. Because of the physical appearance, painful flare-ups and malodorous discharge, this condition may have a negative psychosocial impact on affected individuals [1,2].

The pathogenic mechanisms underlying HS are not fully elucidated, but genetic predisposition, environmental factors, host-microbe interactions, and immune dysregulation seem to be involved in the disease development [3].

Periodontitis is a multifactorial inflammatory disorder caused by dysbiotic microflora and excessive host response, resulting in progressive destruction of the tooth-supporting apparatus. In the course of the disease, the gingival sulcus is deepened to form a periodontal pocket colonized by perio-pathogens [4]. Although more than 700 different types of bacteria reside in the oral cavity, only a small portion of these microorganisms may trigger the destruction of periodontal tissues [5]. Periodonto-pathogenic species were first grouped by Socransky [6] in five colorimetrically coded complexes—green, yellow, orange, red and purple. The particular dental plaque bacteria colonize the gingival sulcus in the specified order via cell-to-cell coaggregation [7]. Oral Streptococci are the dominant species of the oral cavity and the pioneer colonizers of tooth surfaces [8]. Streptococcus species provide additional binding sites for the subsequent deposition of secondary colonizers. The primary as well as secondary colonizers are considered early colonizers and involve green, yellow and violet complexes and *Actinomyces* [9]. The growth of these species groups leads to the proliferation of mainly gram-negative anaerobic bridging and late colonizers. The bridging colonizers form the orange complex and enable the multiplication of late colonizers including the red complex species [10].

The association between periodontitis and various autoimmune skin diseases has been recognized previously [11,12,13,14,15,16]. Several cross-sectional investigations reported a higher incidence of periodontitis and more advanced periodontal disease involvement among patients with psoriasis than among controls. This association was positively correlated with psoriasis severity [11,12]. Moreover, a bi-directional relationship between psoriasis and periodontitis has been suggested, with psoriasis contributing to periodontitis, and vice versa [11,12,17]. Psoriasis has also been demonstrated to be associated with characteristics of salivary microbiota and salivary levels of inflammation-related proteins, which differ from those of individuals with periodontitis and controls [18]. Noteworthy, a significantly higher number of missing teeth and lower radiographic bone level were noticed in psoriasis cases compared to control group [11]. Patients with oral pemphigus vulgaris and mucous membrane pemphigoid appear to be more susceptible to periodontitis, that in turn can potentially induce bullous disorders [19]. Individuals with mucous membrane pemphigoid have more gingival inflammation and worse periodontal status than a control population [13]. Significantly higher values of plaque index, probing depth and clinical attachment level were noticed in pemphigus vulgaris patients than among healthy subjects. Several studies revealed significantly worse periodontal status in patients with lichen planus compared to healthy controls [20,21]. Another study showed that increased plaque and calculus deposits are connected with a significantly higher prevalence of atrophic-erosive gingival lesions in individuals with oral lichen planus [22].

Periodontitis has also been linked with the development of other immune-mediated inflammatory disorders, such as inflammatory bowel diseases (IBD), psoriatic arthritis and rheumatoid arthritis [23,24,25]. Several studies demonstrated a high prevalence of periodontitis in patients with IBD [25,26]. Furthermore, IBD patients harbored higher levels of pathogenic bacteria in inflamed subgingival sites compared to patients with periodontitis [26]. The strict association between rheumatoid arthritis and periodontitis has been revealed. Individuals with rheumatoid arthritis were significantly more frequently diagnosed with periodontitis. This prevalence has been reported to be higher in patients at the earliest stages of the disease and in seropositive subjects [24]. Compelling epidemiological evidence confirms that the risk of periodontal disease is elevated for psoriatic arthritis. Periodontitis severity, as defined by clinical attachment level, was higher in the patients with psoriatic arthritis than in the reference group [12,23].

However, the current knowledge on oral health and periodontal status in HS patients is limited. Recent findings suggested that periodontal disease may be linked with HS [27]. In this study, periodontitis was significantly more frequently diagnosed in patients with HS than among controls. HS and periodontitis seem to share some pathogenic similarities. The IL-23/IL-17 axis plays an essential role in the development and progression of periodontitis as well as HS [28,29]. Several studies provide evidence that HS can be associated with specific alterations in the skin microbiome and toll-like receptors (TLRs) act as important factors in the development of both entities [30,31,32]. Although common inflammatory pathways are implicated in the pathogenesis of these disorders, the exact mechanism of relationship between them is unknown.

The primary objective of the present study was to characterize the composition of periodontal pathogens and evaluate the quantity of salivary microbiota in HS patients. We compared these data to those in patients with periodontitis and healthy controls, to examine the association between HS and periodontitis.

## 2. Materials and Methods

### 2.1. Study Groups

A cross-sectional study was performed from December 2021 until May 2022. Individuals with HS were recruited at the Department of Dermatology, Venereology, and Allergology, Wroclaw Medical University, while individuals with clinical features typical of periodontitis were recruited at the Department of Periodontology, Wroclaw Medical University. A total of 30 healthy controls, 30 HS patients and 30 periodontitis patients were enlisted in the study. The general exclusion criteria were as follows: other systemic diseases, pregnancy, breast-feeding, being under the age of 18, and the use of local and systemic antimicrobials ≤3 months prior to the study baseline. Subjects with HS were excluded if they had periodontitis, and subjects with periodontitis were excluded if they had concomitant HS. The study was approved by the local ethical committee (consent no. 919/2021, date: 26 November 2021). The objective of the study was clarified and written informed consent was received from each participant before the commencement of the study.

### 2.2. Periodontal Evaluation

Periodontal examination was conducted in all patients by a single examiner using the WHO periodontal probe with a probing force of not more than 20 g. The dentition was divided into sextants, and each sextant was examined only if there were two or more teeth present and not indicated for extraction. Diagnosis of periodontitis was established after a complete periodontal inspection using probing depth and attachment loss evaluation. In the current study, periodontitis was defined as the presence of interdental clinical attachment loss (CAL) at two or more non-adjacent teeth or the presence of oral or buccal CAL no less than 3 mm with pocketing >3 mm at ≥two teeth [33]. The severity and extent of the management required were assessed using the staging (stage I: initial periodontitis; stage II: moderate periodontitis; stages III and IV: severe periodontitis), while the progression rate of the periodontitis was assessed using the grading (grade A: slow; grade B: moderate; grade C: rapid rate of progression) [34].

### 2.3. Dermatological Evaluation

All participants in the study were evaluated by a dermatologist for systemic status, cutaneous and mucosal lesions. Patients diagnosed with HS without any other concomitant skin, periodontal or systemic disorder were enrolled in the HS group. In addition, patients from periodontitis and control groups were excluded if they were diagnosed with any chronic cutaneous or systemic disease. HS severity stage was assessed in patients from HS group using the Hurley staging system and International Hidradenitis Suppurativa Severity Score System (IHS4). In addition, after establishment of the IHS4 score, the subjects were subsequently divided into 3 groups (mild, moderate, and severe disease). Cut-off points were employed for mild (≤3 points), moderate (>3 and ≤10 points) and severe HS (>10 points) [35,36].

### 2.4. Subgingival Plaque Sample Collection

The deepest periodontal pocket was identified for every study subject during the clinical periodontal examination and subgingival bacterial plaque samples were obtained. Before sampling, the supragingival bacterial plaque was cleaned, and then each tooth was isolated with cotton rolls and dried thoroughly with an air syringe. A sterile paper point included in the diagnostic kit was introduced inside each gingival sulcus for 20 s using tweezers. The samples were loaded into test tubes and shipped to the MIP International Pharma Research GmbH Laboratory located in Germany, where sample processing was performed.

### 2.5. Microbiological Analysis

PET Test^®^ plus is a CE-certified medical device, that is manufactured by MIP Pharma GmbH. The exact protocol for the microbiological examination procedure is confidential to the company. Sample analysis was conducted using a real-time polymerase chain reaction (RT-PCR). The PCR-based test allowed detection and quantification of nine crucial perio-pathogens (*Porphyromonas gingivalis*, *Treponema denticola*, *Tannerella forsythia*, *Prevotella intermedia*, *Peptostreptococcus micros*, *Fusobacterium nucleatum*, *Eubacterium nodatum*, *Capnocytophaga gingivalis* and *Aggregatibacter actinomycetemcomitans*) in the study’s samples. Free strand sections of DNA were obtained from lysed bacterial cells and were subsequently subjected to amplification and hybridization using fluorescence-stained starters characteristic of particular periodontal pathogens. The quantitative analysis of the samples was carried out with a reader that measures fluorescence intensity compared to that in reference specimens. According to information from the manufacturer, the threshold determination for all analyzed perio-pathogens was approximately 10^3^ bacteria.

### 2.6. Statistical Analysis

Statistical analysis of the obtained results was performed with the use of the IBM SPSS Statistics v. 26 (SPSS INC., Chicago, IL, USA) software. All data was assessed for normal or abnormal distribution. The minimum, maximum, mean, and standard deviation were calculated. Differences in quantitative variables between two groups, depending on the normality, were evaluated using the t-Student test or Mann–Whitney U test. Correlation between quantitative data were assessed, depending on normality, with Pearson’s and Spearman’s correlations. For qualitative data, the Chi-squared test was used. Differences in number of copies of perio-pathogens between more than two groups were assessed, depending on the normality, with the use of the one-way analysis of variance on ranks (ANOVA) or Kruskal-Wallis test with the adjustment according to the Bonferroni correction. A two-sided *p* of less than 0.05 was considered statistically significant.

## 3. Results

The HS, periodontitis and control groups consisted of 12 males and 18 females aged 36.1 ± 11.64 (range, 20–75) years, 12 males and 18 females aged 41.5 ± 9.78 (range, 27–53) years, and 10 males and 20 females aged 33.5 ± 7.40 (range, 20–52) years, respectively.

The severity of the disease in the HS group in the majority of patients (14 patients, 46.6%) was assessed as Hurley stage II, in eight patients (26.7%) as Hurley stage I, and in eight patients (26.7%) as Hurley stage III. As for IHS4, 12 patients (40.0%) presented with moderate HS, 12 patients (40.0%) presented with severe HS, and six patients (20.0%) presented with mild HS. The mean IHS4 score among HS patients was assesed as 19 ± 22.28 points.

The periodontitis stage in the periodontits group in the majority of individuals (14 patients, 46.6%) was assessed as stage IV, in 12 patients (40.0%) as stage III, in three patients (10.0%) as stage II, and in one patient (3.4%) as stage I. The most frequent grade was grade B (17 patients, 56.7%), while grade C was present in 13 individuals (43.3%), and none of the patients with periodontitis presented with grade A.

**Figure 1:**
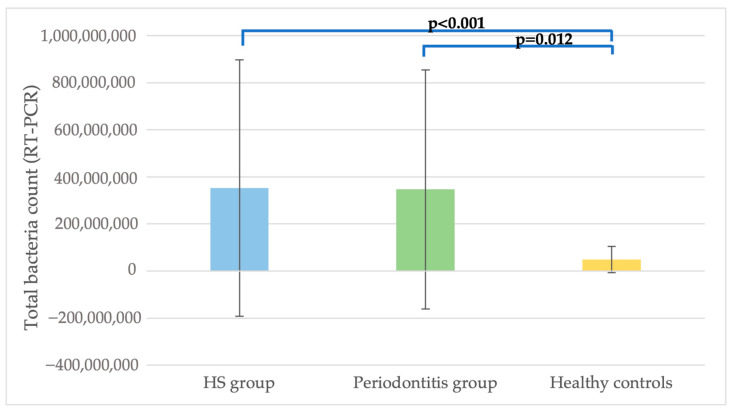
A comparison of the average copy number of total bacteria in the HS, periodontitis and control groups. HS: hidradenitis suppurativa, RT-PCR: real-time polymerase chain reaction.

The average copy-count number of total bacteria was significantly higher in the HS and periodontitis samples than in the control samples (*p* = 0.04) (Figure 1).

**Figure 2:**
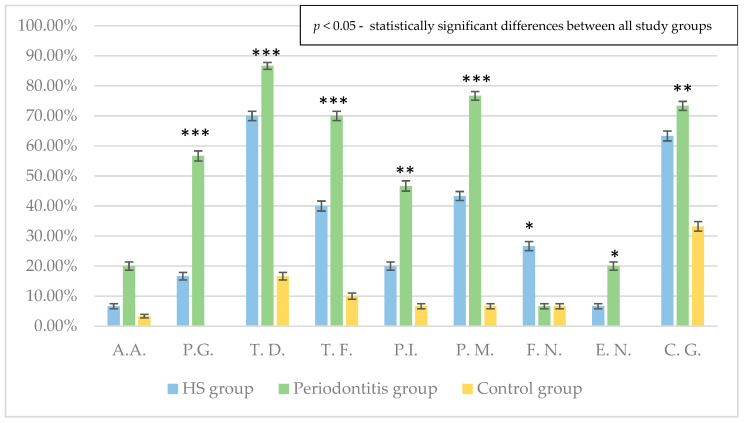
Percentages of patients in the HS, periodontitis and control groups with particular -. * *p*  <  0.05, ** *p*  <  0.01, *** *p*  <  0.001, HS: hidradenitis suppurativa, A.A.: *Aggregatibacter actinomycetemcomitans*, P.G.: *Porphyromonas gingivalis*, T.D.: *Treponema denticola*, T.F.: *Tannerella forsythia*, P.I.: *Prevotella intermedia*, P.M.: *Peptostreptococcus micros*, F.N.: *Fusobacterium nucleatum*, E.N.: *Eubacterium nodatum*, C.G.: *Capnocytophaga gingivalis*.

Statistically significant differences in the prevalence of periopathogens between groups were found for all bacterial species except *A. actinomycetemcomitans* (Figure 2).

Most perio-pathogenic bacteria were more frequently detected in the subgingival plaque both in HS and periodontitis patients than in healthy controls (Table 1 and Table 2).

*T.denticola* was the most frequently isolated pathogen in individuals with HS (70%) and periodontitis (86.7%), whereas among controls *C. gingivalis* was the most common microorganism (33.2%). The microbiological results revealed significant differences in the prevalence of periopathogens between HS and periodontitis groups concerned the following species: *P.gingivalis*, *T. forsythia*, *P. intermedia*, *P. micros* and *F. nucleatum*. The first four above-mentioned species were significantly more common among periodontitis patients, while *F. nucleatum* was identified more frequently in HS individuals (Table 3).

**Figure 3:**
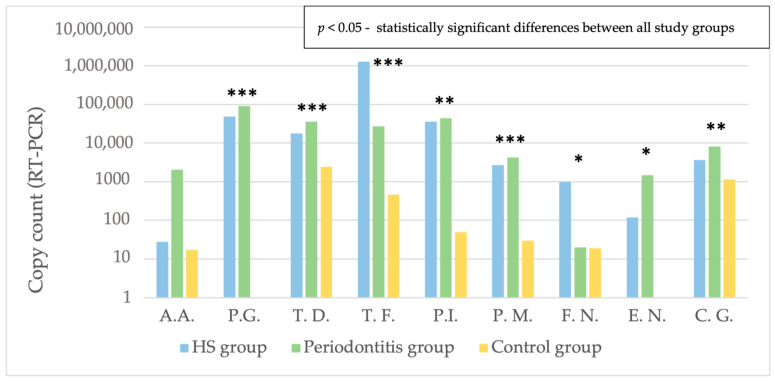
A comparison of the average copy number of perio-pathogens in the HS, periodontitis and control groups. * *p*  <  0.05, ** *p*  <  0.01, *** *p*  <  0.001, HS: hidradenitis suppurativa, A.A.: *Aggregatibacter actinomycetemcomitans*, P.G.: *Porphyromonas gingivalis*, T.D.: *Treponema denticola*, T.F.: *Tannerella forsythia*, P.I.: *Prevotella intermedia*, P.M.: *Peptostreptococcus micros*, F.N.: *Fusobacterium nucleatum*, E.N.: *Eubacterium nodatum*, C.G.: *Capnocytophaga gingivalis*.

The average copy number of all periopathogenic bacteria except *A.actinomycetemcomitans* differed significantly between groups (Figure 3).

The average copy-count number of *T. denticola*, *T. forsythia*, *P. micros* and *C. gingivalis* was significantly higher in both periodontitis and HS groups compared to controls (Table 1 and Table 2). *P.gingivalis* and *P. micros* species were expressed at higher level in periodontitis patients than in HS patients (Table 3).

Noteworthy, there was no correlation between total bacterial count as well as quantity of particular periopathogens and HS severity assessed both with Hurley and IHS4 scales in the HS group. Similarly, the duration of the disease was not correlated with the copy number of periodontal pathogens.

## 4. Discussion

Dental plaque plays a crucial role in the patho-etiology of periodontal disease [37]. Many studies in the literature focus on whether various autoimmune disorders contribute to periodontitis, and conversely, whether periodontitis may influence systemic diseases’ development and spread [25,38,39,40]. The current knowledge on periodontal status in HS is limited. Recent data showed a higher prevalence of periodontitis among HS patients, suggesting possible links between these entities [27].

Our study revealed that HS patients, similarly to periodontitis patients, tended to be more frequently infected with perio-pathogenic bacteria compared to orally healthy controls. Furthermore, the total bacteria count and the DNA copies number of a large portion of perio-pathogenic species were significantly higher in HS and periodontitis groups than among controls. These findings suggest that HS may influence oral homeostasis and HS individuals might be more prone to periodontal disease. On the other hand, periodontal pathogens may also enhance HS progression by promoting an inflammatory milieu.

Bacteria genera that were increased in occurrence in HS patients without periodontal disease compared to orally healthy controls were *P.gingivalis*, *T. denticola*, *T. forsythia*, *P. micros*, *F. nucleatum* and *C. gingivalis.* The three first species mentioned above form the red complex that acts as a pathogenic consortium in periodontitis. *P. micros and F. nucleatum* constitute the orange complex, while *C. gingivalis* belongs to the green complex [6].

*P.gingivalis* is a gram-negative, non-motile, anaerobic bacterium of the oral cavity [41]. This bacterial species has been recognized for its role in the regulation of distant inflammatory responses connected with chronic conditions and autoimmune diseases [42]. Several investigations reported that *P.gingivalis* exposure might be linked to systemic diseases such as rheumatoid arthritis (RA), inflammatory bowel diseases, diabetes mellitus and atherosclerosis [43,44,45,46]. *P. gingivalis* has been referred to as a master of immune subversion, utilizing unique and intricate sabotage techniques to evade and weaken the host’s immune system [47]. It modifies the functions of various innate immune signaling cascade components such as the complement system, toll-like receptors (TLRs), macrophages, neutrophils, dendritic cells, and T cells [48]. The lipopolysaccharide (LPS) of *P. gingivalis* exhibits two isoforms responsible for the dual inflammatory response via TLRs modulation. The penta-acylated LPS induces TLR4 and TLR2 when tetra-acylated LPS is a TLR4 antagonist and TLR2 an agonist [49]. *P. gingivalis* triggers the release of IL-1, IL-6, IL-8, and TNF-α, acting by TLR4/TLR2 in host cells [50]. IL-1β production, maturation, and secretion are tightly modulated by TLR signaling as well as inflammasome activation [51]. *P. gingivalis* stimulated innate immune cells by the nucleotide-binding domain-like receptor protein 3 (NLRP3) inflammasome. The NLRP3 inflammasome and the following response from the IL-1 family might play an important role in periodontal disease triggered via *P. gingivalis* challenge through sustained inflammatory milieu [52,53].

*T. denticola* is a motile oral spirochete, while *T. forsythia* is a non-motile, rod-shaped microorganism [54,55,56,57]. A recent study examined the correlation between systemic lupus erythematosus (SLE) disease activity and severity and perio-pathogenic bacteria and reported that abundance of *T. denticola* and *T. forsythia* was increased in SLE-active periodontal sites compared to that of SLE-inactive and controls [58]. Moreover, serum antibody titers against *T. denticola*, *P. gingivalis*, *A. actinomycetemcomitans* and *C. ochracea* species were positively correlated with anti-dsDNA titers and reduced complement levels in SLE patients [59]. The study on the association of RA with periodontopathic bacterial infection revealed that serum and synovial fluid antibodies to *T. forsythia*, *P. gingivalis*, *P. intermedia*, and *Prevotella melaninogenica* were detected in RA patients [60]. Several studies showed that *T. denticola* enhances the synthesis of various cytokines, including IL-1β, IL-6, IL-8, and TNF-α from different cell types [61,62,63,64]. Conversely, it has also been demonstrated that *T. denticola* suppresses IL-8 production [65].

The gram-positive anaerobic coccus *P. micros* was found to be above the detection threshold in RA patients [66]. This bacterium has been reported to induce intracellular signaling mechanisms, resulting in an increased production of pro-inflammatory cytokines such as TNF- α, IL-1beta and IL-6 and chemokines through macrophages [6,67,68]. Noteworthy, *C. gingivalis*, which is a gram-negative rod, has also been shown to induce the release of IL-6 [69,70].

*F. nucleatum* was the only perio-pathogenic bacterium that was more prevalent among HS individuals than among periodontitis patients. This gram-negative pathogen has been reported to be associated with many systemic diseases, including atherosclerosis, adverse pregnancy outcomes, polycystic ovary syndrome, colorectal cancer and RA [71,72,73,74,75]. *F. nucleatum* induces a spectrum of host immune responses and acts as a potent stimulator of inflammatory cytokines. Chronic local infection of *F. nucleatum* initiates the up-regulation of inflammatory pathways. It stimulates various cytokines, such as IL-6, IL-8 and TNF-α [76,77]. Moreover, chronic inflammation caused by *F. nucleatum* contributes to the progression of various systemic diseases via modulation of TLRs and promoting CD4+ T cell proliferation and differentiation in Th1 and Th17 [78,79,80].

The role of dysbiosis in HS development is not fully elucidated [32]. The profile of skin microbiota changes with the progression of the disease, but it remains to be determined if this is a primary or secondary event [81]. The preponderance of *Staphylococcus* was noted in early HS lesions, while gram-negative anaerobic bacteria such as *Porphyromonas* and *Prevotella* were predominantly identified in HS tunnels and chronic suppurating lesions [81,82]. Patients affected with HS present elevated levels of pro-inflammatory cytokines, including TNF-α, IL-1β and IL-6, which are also implicated in perio-pathogens-induced immune responses [2,50,67,68]. The expression of IL-1β was significantly increased in the HS lesional and perilesional skin compared with uninvolved HS skin or healthy control skin [83]. TNF-α inhibitors have demonstrated a significant efficacy in individuals with moderate to severe HS [84]. Studies revealed increased serum IL-6 levels in Hurley II and III HS individuals, indicating that IL-6 may participate in the development of HS [85,86]. Moreover, TLRs and NLRP3 inflammasome, which are involved in periodontal bacteria pathogenicity, play also a role in the pathogenesis of HS [87,88].

Some limitations apply to the study. The first concerns the relatively low number of included individuals with HS, resulting from the single-center setting. Nevertheless, as significant differences in the composition of subgingival microbiota were noted, the number of participants might have been sufficient. Furthermore, the lack of follow-up examinations is another limitation and should be considered in future studies.

## 5. Conclusions

In conclusion, data from the present investigation prove that HS and periodontitis patients share similarities in their subgingival microbiome composition. Further studies are needed to understand the relationship between oral dysbiosis, immune dysregulation and HS pathogenesis. Future investigation should incorporate larger patient populations to confirm the initial results. In addition, the evaluation of the relationship between HS and periodontitis using the selected markers of inflammation would allow an understanding of interlinkage mechanisms.

## Figures and Tables

**Table 1:** The prevalence and the copy number of oral microorganisms in patients with periodontitis and controls.

	Number (%) of Infected Individuals			Average Copy Number of Bacteria (RT-PCR) (Mean ± SD)		
Periopathogens Tested	Patients with Periodontitis (*n* = 30)	Controls (*n* = 30)	*p*	Patients with Periodontitis	Controls	*p*
Total bacteria	30 (100%)	30(100%)	NA	3.5 × 10^8^ ± 5.1 × 10^8^	4.9 × 10^7^ ± 5.6 × 10^7^	<0.001
*Aggregatibacter actinomycetemcomitans*	6 (20.0%)	1 (3.3%)	0.044	2.0 × 10^3^ ± 7.7 × 10^3^	18 ± 97	NS
*Porphyromonas gingivalis*	17 (56.7%)	0 (0.0%)	<0.001	9.1 × 10^4^ ± 3.5 × 10^5^	0	<0.001
*Treponema denticola*	26 (86.7%)	5 (16.7%)	<0.001	3.6 × 10^4^ ± 8.4 × 10^4^	2.4 × 10^3^ ± 9.7 × 10^3^	<0.001
*Tanerella forsythia*	21 (70.0%)	3 (10.0%)	<0.001	2.7 × 10^4^ ± 6.5 × 10^4^	4.6 × 10^2^ ± 2.2 × 10^3^	<0.001
*Prevotella intermedia*	14 (46.7%)	2 (6.7%)	<0.001	4.8 × 10^4^ ± 1.3 × 10^5^	50 ± 2.6 × 10^2^	0.001
*Peptostreptococcus micros*	23 (76.7%)	2 (6.7%)	<0.001	4.2 × 10^3^ ± 6.9 × 10^3^	30 ± 1.2 × 10^2^	<0.001
*Fusobacterium nucleatum*	2 (6.7%)	2 (6.7%)	NS	20 ± 79	3.6 × 10^2^ ± 19	NS
*Eubacterium nodatum*	6 (20.0%)	0 (0.0%)	0.010	1.5 × 10^3^ ± 6.4 × 10^3^	0	0.021
*Capnocytophaga gingivalis*	22 (73.3%)	10 (33.2%)	0.002	8.1 × 10^3^ ± 1.5 × 10^4^	1.2 × 10^3^ ± 4.3 × 10^3^	0.001

NS: Not statistically significant, NA: Not applicable, SD: standard deviation.

**Table 2:** The prevalence and the copy number of oral microorganisms in patients with HS and controls.

	Number (%) of Infected Individuals			Average Copy Number of Bacteria (RT-PCR) (Mean ± SD)		
Periopathogens Tested	Patients with HS (*n* = 30)	Controls (*n* = 30)	*p*	Patients with HS	Controls	*p*
Total bacteria	30 (100%)	30(100%)	NA	3.5 × 10^8^ ± 5.4 × 10^8^	4.9 × 10^7^ ± 5.6 × 10^7^	0.012
*Aggregatibacter actinomycetemcomitans*	2 (6.7%)	1 (3.3%)	NS	28 ± 1.2 × 10^2^	18 ± 97	NS
*Porphyromonas gingivalis*	5 (16.7%)	0 (0.0%)	0.020	4.8 × 10^4^ ± 1.6 × 10^5^	0	NS
*Treponema denticola*	21 (70.0%)	5 (16.7%)	<0.001	1.8 × 10^4^ ± 2.7 × 10^4^	2.4 × 10^3^ ± 9.7 × 10^3^	<0.001
*Tanerella forsythia*	12 (40.0%)	3 (10.0%)	0.007	1.3 × 10^6^ ± 6.8 × 10^6^	4.6 × 10^2^ ± 2.2 × 10^3^	0.042
*Prevotella intermedia*	6 (20.0%)	2 (6.7%)	NS	3.6 × 10^4^ ± 1.2 × 10^5^	50 ± 2.6 × 10^2^	NS
*Peptostreptococcus micros*	13 (43.3%)	2 (6.7%)	0.001	2.7 × 10^3^ ± 1.2 × 10^4^	30 ± 1.2 × 10^2^	0.033
*Fusobacterium nucleatum*	8 (26.7%)	2 (6.7%)	0.038	9.8 × 10^2^ ± 4.0 × 10^3^	3.6 × 10^2^ ± 19	NS
*Eubacterium nodatum*	2 (6.7%)	0 (0.0%)	NS	1.2 × 10^2^ ± 4.8 × 10^2^	0	NS
*Capnocytophaga gingivalis*	19 (63.3%)	10 (33.2%)	0.020	3.7 × 10^3^ ± 1.0 × 10^4^	1.2 × 10^3^ ± 4.3 × 10^3^	0.032

NS: Not statistically significant, NA: Not applicable, SD: standard deviation, HS: hidradenitis suppurativa.

**Table 3:** The prevalence and the copy number of oral microorganisms in patients with HS and periodontitis.

	Number (%) of Infected Individuals			Average Copy Number of Bacteria (RT-PCR) (Mean ± SD)		
Periopathogens Tested	Patients with HS (*n* = 30)	Patients with Periodontitis (*n* = 30)	*p*	Patients with HS	Patients with Periodontitis	*p*
Total bacteria	30 (100%)	30 (100%)	NA	3.5 × 10^8^ ± 5.4 × 10^8^	3.5 × 10^8^ ± 5.1 × 10^8^	NS
*Aggregatibacter actinomycetemcomitans*	2 (6.7%)	6 (20.0%)	NS	28 ± 1.2 × 10^2^	2.0 × 10^3^ ± 7.7 × 10^3^	NS
*Porphyromonas gingivalis*	5 (16.7%)	17 (56.7%)	0.001	4.8 × 10^4^ ± 1.6 × 10^5^	9.1 × 10^4^ ± 3.5 × 10^5^	0.003
*Treponema denticola*	21 (70.0%)	26 (86.7%)	NS	1.8 × 10^4^ ± 2.7 × 10^4^	3.6 × 10^4^ ± 8.4 × 10^4^	NS
*Tanerella forsythia*	12 (40.0%)	21 (70.0%)	0.02	1.3 × 10^6^ ± 6.8 × 10^6^	2.7 × 10^4^ ± 6.5 × 10^4^	NS
*Prevotella intermedia*	6 (20.0%)	14 (46.7%)	0.028	3.6 × 10^4^ ± 1.2 × 10^5^	4.8 × 10^4^ ± 1.3 × 10^5^	NS
*Peptostreptococcus micros*	13 (43.3%)	23 (76.7%)	0.008	2.7 × 10^3^ ± 1.2 × 10^4^	4.2 × 10^3^ ± 6.9 × 10^3^	0.033
*Fusobacterium nucleatum*	8 (26.7%)	2 (6.7%)	0.038	9.8 × 10^2^ ± 4.0 × 10^3^	20 ± 79	NS
*Eubacterium nodatum*	2 (6.7%)	6 (20.0%)	NS	1.2 × 10^2^ ± 4.8 × 10^2^	1.5 × 10^3^ ± 6.4 × 10^3^	NS
*Capnocytophaga gingivalis*	19 (63.3%)	22 (73.3%)	NS	3.7 × 10^3^ ± 1.0 × 10^4^	8.1 × 10^3^ ± 1.5 × 10^4^	NS

NS: Not statistically significant, NA: Not applicable, SD: standard deviation, HS: hidradenitis suppurativa.

## Data Availability

The datasets generated and analyzed in the current study are available from the corresponding author upon reasonable request.
